# Knowledge-guided adaptive educational support for traditional Chinese medicine using large language models and knowledge graphs: integrating Confucian educational principles

**DOI:** 10.3389/fmed.2026.1943401

**Published:** 2026-09-03

**Authors:** Jili Hu, Yuchen Duan, Qingqing Zhou, Denggao Zheng, Yu Li, Chi Qin, Yu Liu, Hongxing Kan, Fan Xu

**Affiliations:** 1 School of Medical Information Engineering, Anhui University of Chinese Medicine, Hefei, China; 2 Center for Xin'an Medicine and Modernization of Traditional Chinese Medicine of IHM, Anhui University of Chinese Medicine, Hefei, China; 3 School of Pharmacy, Anhui University of Chinese Medicine, Hefei, China; 4 Anhui Provincial Key Laboratory of Chinese Medicinal Formula, Hefei, China

**Keywords:** adaptive educational support, intelligent tutoring system, knowledge graphs, large language models, traditional Chinese medicine

## Abstract

**Introduction:**

The Confucian principle of “teaching students in accordance with their aptitude” remains challenging to implement at scale in Traditional Chinese Medicine (TCM) education. We developed an Intelligent TCM Tutoring System integrating a large language model (LLM) with a domain-specific knowledge graph (KG) derived from the Golden Mirror of Medicine and evaluated its system performance and short-term educational outcomes.

**Methods:**

System outputs were rated by five TCM teachers. In a three-group pedagogical experiment, 125 student volunteers were randomly allocated to self-directed learning, LLM-assisted learning, or learning with the integrated system, and 102 questionnaires were included in the final analysis.

**Results:**

The integrated system received higher expert ratings than the LLM baseline without KG integration in instructional content generation, intelligent question answering, and automated test generation and grading. Students using the integrated system achieved significantly higher immediate post-test scores than both comparison groups and reported more favorable post-session learning experience ratings in relevant comparisons.

**Discussion:**

These findings provide preliminary evidence that an LLM-KG system can enhance immediate learning outcomes and learning experiences in this educational setting.

## 1 Introduction

Traditional Chinese Medicine (TCM) refers to a traditional medical system originating in China that emphasizes a holistic approach and diagnosis and treatment based on syndrome differentiation. As an integral component of Chinese culture, it encompasses theoretical and practical knowledge developed through centuries of clinical practice (1, 2). The effective transmission of this complex knowledge is paramount for its sustained development. Historically, TCM education centered on an apprenticeship model, where masters imparted theoretical knowledge and clinical experience through personalized instruction (3, 4). This approach is rooted in the Confucian principle of “teaching students in accordance with their aptitude” (*Yin Cai Shi Jiao*), which advocates for instruction tailored to individual learner differences (5, 6). This philosophy aligns with modern learner-centered teaching theories (7), which prioritize student initiative and development, underscoring its cross-cultural educational value, especially in a specialized field like TCM (8, 9).

However, the traditional apprenticeship model lacked standardization for large-scale talent cultivation. Consequently, university-based education became the primary training pathway in China to ensure scale and uniformity (3). Despite its systematic approach, the expansion of modern higher TCM education has led to challenges such as faculty shortages and resource constraints. This often results in homogenized training, hindering the implementation of personalized instruction and failing to cater to individual student needs (10). Students face a vast, fragmented curriculum presented in classical Chinese, making core concepts difficult to grasp (11). The predominant teacher-centered pedagogical model is often inefficient, while existing formative assessments provide feedback with significant delays and lack specificity (12). As a result, instructors struggle to track student progress dynamically and provide the necessary personalized guidance.

To address these challenges, integrating modern information technology is crucial for enabling intelligent teaching and personalized learning (13, 14). The rapid development of artificial intelligence (AI), particularly large language models (LLMs), offers powerful tools for personalized education through advanced language understanding and generation capabilities (15, 16). Concurrently, Knowledge Graphs (KGs) provide a robust method for structuring the vast, complex knowledge of TCM into a coherent semantic network, facilitating knowledge visualization and management (17).

This study proposes that integrating KGs with LLMs can create a powerful synergy for TCM education. This approach allows a system to leverage semantic relationships for more precise and logically structured responses to complex queries (18). It can visualize the knowledge system and generate learning resources in response to selected topics and user queries, thereby supporting adaptive educational interactions. Inspired by the principle of “teaching students in accordance with their aptitude,” we designed a functional framework for an Intelligent TCM Tutoring System, as illustrated in Figure 1. We investigate this approach through a case study of the *Golden Mirror of Medicine* (19), a foundational Qing dynasty text renowned for its systematic and comprehensive content (20, 21). Despite its importance, its classical language and abstract semantics present significant learning challenges for modern students, necessitating innovative pedagogical tools (22).

**Figure 1:**
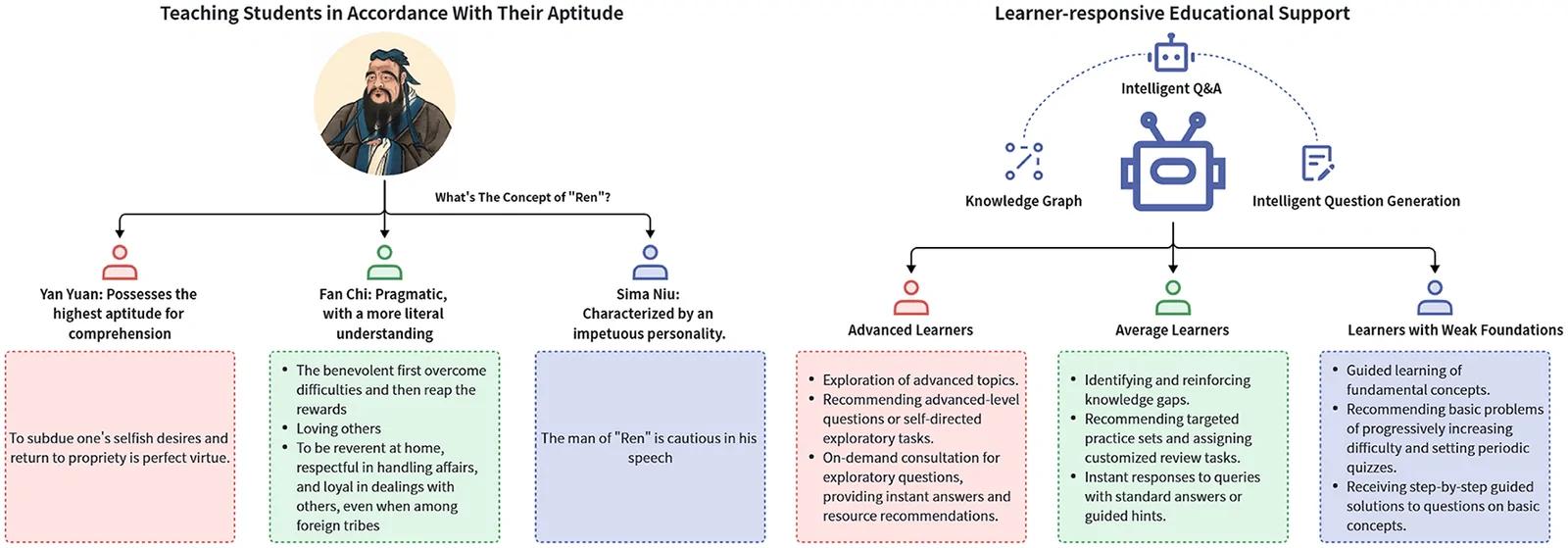
Conceptual framework of the Intelligent TCM Tutoring System. This schematic maps the Confucian principle of “teaching students according to their aptitude” onto learner-responsive system support. The left panel illustrates how Confucius provided differentiated explanations of “Ren” (benevolence) to Yan Yuan, Fan Chi, and Sima Niu according to their characteristics and levels of understanding. The right panel shows how the system combines a knowledge graph, intelligent question answering, and intelligent question generation to support advanced learners, average learners, and learners with weak foundations through differentiated learning resources and activities. Adapted from Unknown artist. Portrait of Confucius at Leisure, Ming dynasty (1368–1644), Confucius Museum, Qufu, China. Photograph by Gary Todd. Creative Commons CC0 1.0 Universal (CC0 1.0). The source image was modified for integration into this figure.

Therefore, this study aims to develop and evaluate an Intelligent TCM Tutoring System that integrates LLMs and KGs to provide adaptive educational support. The main contributions are:

Constructing a systematic *Golden Mirror of Medicine* KG as a foundation for the digital transformation of TCM educational resources.

Proposing an LLM-KG integrated teaching method designed to enhance instructional efficiency and student learning outcomes in TCM.

Evaluating the method's pedagogical performance through a classroom experiment, using student performance and subjective feedback to assess its practicality and effectiveness.

This research not only offers a novel approach to the digitalization of TCM education but also presents a transferable model for applying integrated LLM and KG technologies in other educational domains. It explores how the principle of “teaching students in accordance with their aptitude” can inform a modern digital framework for learner-responsive educational support.

## 2 Related work

### 2.1 Applications of large language models in education

In recent years, significant progress has been made in the application of LLMs in education, where they have demonstrated immense potential, particularly in supporting adaptive and learner-responsive educational experiences. LLMs can provide customized knowledge generation, answer queries, and recommend learning paths tailored to learners' proficiency levels, interests, and needs, thereby facilitating a paradigm shift in education from “one-size-fits-all instruction” to “personalized guidance.” For instance, Mihaljević et al. (23) utilized an LLM-supported tool in a software development course, which enhanced students' efficiency in learning programming skills and significantly improved their comprehension and completion of programming tasks, demonstrating the model's adaptive support for students at varying proficiency levels. Spina et al. (24) leveraged GPT-4 to simplify medical literature on glaucoma, which not only enhanced patient comprehension but also enabled audiences with diverse educational backgrounds to effectively access key information, showcasing the LLM's capacity to tailor content to individual cognitive differences. Guo et al. (25) integrated an LLM into middle school science and engineering practice courses and found that, compared to traditional methods, this integrated approach significantly enhanced students' individualized development across multiple dimensions, including scientific knowledge, attitudes toward technology, and problem-solving skills. Wang et al. (26) validated the feasibility of using ChatGPT as a standardized patient simulation tool, demonstrating that an optimized LLM can personalize roles and feedback for different learning scenarios, thus enhancing the interactivity and realism of medical education. Ye et al. (27), by optimizing the LLM's reasoning chain, provided personalized explanations for mathematics learners, effectively improving learning outcomes, comprehension, and engagement, further demonstrating the value of LLMs in promoting deeply personalized learning. The Khanmigo system (28) launched by Khan Academy does not directly provide answers but instead guides learners, acting as a tutor, to explore solutions independently, promoting critical thinking and fostering independent problem-solving abilities, which aligns with the student-centered philosophy of personalized education.

Taken together, these studies show that LLMs can support educational content adaptation, guided inquiry, simulated-patient interaction, and personalized explanations across diverse learning contexts. However, these applications are primarily general-purpose or task-specific. In medicine, persistent concerns regarding domain accuracy, reliability, and traceability constrain their use for specialized instruction. Evidence in TCM education remains particularly limited, especially regarding the grounding of an LLM in a structured classical TCM knowledge source and its application across multiple instructional tasks. These gaps highlight the need for integrating LLMs with explicit domain knowledge structures to support more reliable and context-specific educational applications.

### 2.2 Role of knowledge graphs in education

Knowledge graphs have been widely applied in the field of education. Hou et al. (29) proposed a personalized learning path planning method based on KGs, which effectively enhanced the consistency and relevance of recommended learning paths and improved learners' learning efficiency. Wei et al. (30) investigated a KG-based recommender system designed to address the issues of information overload and knowledge fragmentation in online education; their experimental results indicated that the system improved students' interest and knowledge retention rates. Zhong et al. (31) constructed a recommendation model using multi-task feature learning, which embeds the KG into tasks and utilizes a cross-compress unit to build high-order connections between latent features and entities across tasks, thereby recommending professional music course teaching resources. Ramazanova et al. (32) studied the use of a heterogeneous KG to build a recommender system. They employed multilingual semantic similarity algorithms and natural language processing (NLP) techniques to analyze and match skills. This approach provided personalized course and job recommendations intended to bridge the gap between educational curricula and the changing demands of the labor market. Lu and Li (33) proposed a delivery method for Chinese language course resources based on KGs and similarity algorithms, introducing a dual-delivery module that effectively reduced the similarity among delivered data, thereby improving delivery efficiency.

Collectively, KG-based studies demonstrate benefits for learning-path planning, resource recommendation, semantic organization, and retrieval. However, many existing KG-based educational applications primarily focus on resource selection and delivery, with relatively limited exploration of generative question answering, assessment, grading, and coordinated support across teaching stages. Applications to classical TCM knowledge are also scarce. Thus, although KGs can organize complex educational knowledge, their use as a domain-grounding layer for interactive and evaluative functions in TCM education remains insufficiently examined.

### 2.3 Synergistic integration of LLMs and KGs in education

The integration of LLMs and KGs combines the generative capabilities of LLMs with the structured knowledge representation of KGs. Prior studies have reported improvements in educational chatbot accuracy and responsiveness (34), cross-source question answering (35), recommendation under cold-start and data-scarcity conditions (36), educational-content review (37), and multimodal question answering for a TCM course (38). These findings indicate that structured knowledge can improve the grounding and task specificity of LLM-supported educational applications.

Nevertheless, the reviewed studies generally address individual functions and provide limited evidence on systems that combine a human-validated domain KG with an end-to-end instructional workflow encompassing content generation, question answering, assessment, grading, and feedback. They also provide limited within-study evidence comparing such an integrated system with the same underlying LLM without KG support. To address these gaps, this study constructs a human-validated KG from the *Golden Mirror of Medicine*, integrates it with an LLM across the instructional workflow, and evaluates both expert-rated system performance and students' immediate learning outcomes and post-session experiences. This design enables a context-specific evaluation of how KG grounding influences the reliability and educational utility of LLM-based support in classical TCM education.

## 3 Materials and methods

### 3.1 Construction of knowledge graph

#### 3.1.1 Data source

This study investigates the construction, application, and effectiveness of the Intelligent TCM Tutoring System through a case study of the *Golden Mirror of Medicine*. The data are drawn from the People's Medical Publishing House edition, a comprehensive collection of 90 volumes (1.766 million Chinese characters) that systematically organizes TCM knowledge into 15 medical texts. This version was selected for its high academic authority, ensured by expert review and a reputable publisher, and its completeness in covering the core content of the original work, thus providing a reliable and systematic data foundation.

#### 3.1.2 Architecture design of knowledge graph

The architectural design of our *Golden Mirror of Medicine* KG is crucial for its pedagogical application in TCM courses. Adopting a top-down approach, we defined the KG's entity types and relations based on the *Health informatics—Semantic network framework of traditional Chinese medicine language system* (39) to ensure its semantic accuracy and alignment with TCM educational needs.

The KG architecture encompasses core entity types such as Diseases, Symptoms, Formulas, Medicinals, and Administration Methods. These entities are interconnected through several key relations. Hierarchical relations, represented by includes, structure the text's contents, chapters, and sections. Treatment-related connections are established through Formula and Herbs relations, which link diseases or texts to their corresponding formulas and constituent medicinals. Furthermore, Symptoms relations serve a diagnostic purpose by connecting diseases to their clinical manifestations, while explanatory relations like Annotation, Note, and Collected Annotation link main texts to their various commentaries.

This structured representation is designed to facilitate a deeper understanding of TCM's complex theoretical system. The KG not only visualizes knowledge but also supports in-depth learning through powerful query capabilities, enabling instructors and students to explore the intricate relationships between concepts like diseases and treatments, thereby enhancing both teaching and learning.

#### 3.1.3 Construction process of knowledge graph

The construction process of our *Golden Mirror of Medicine* KG involved three main stages: automated extraction, expert validation, and data storage. First, we employed an LLM to perform an initial extraction of entities and relations from the source text, forming a foundational information framework. Second, to ensure accuracy and reliability, we implemented a rigorous expert validation protocol. The text was segmented by book, and for each segment, two TCM professionals independently reviewed the LLM-extracted data. A cross-checking methodology was used to resolve discrepancies, reduce subjective bias, and standardize the KG's content. A key principle during this stage was to preserve the original classical expressions from the *Golden Mirror of Medicine*, rather than modernizing them, to foster a deeper understanding among students of the classic text. Finally, the validated relational triples were exported into node and relation CSV files and subsequently batch-imported into a Neo4j graph database using the neo4j-admin utility. This choice of database facilitates efficient storage, rapid retrieval, and intuitive visualization of the complex knowledge network, enhancing the KG's utility as a pedagogical tool. A partial view of the resulting KG is shown in Figure 2.

**Figure 2:**
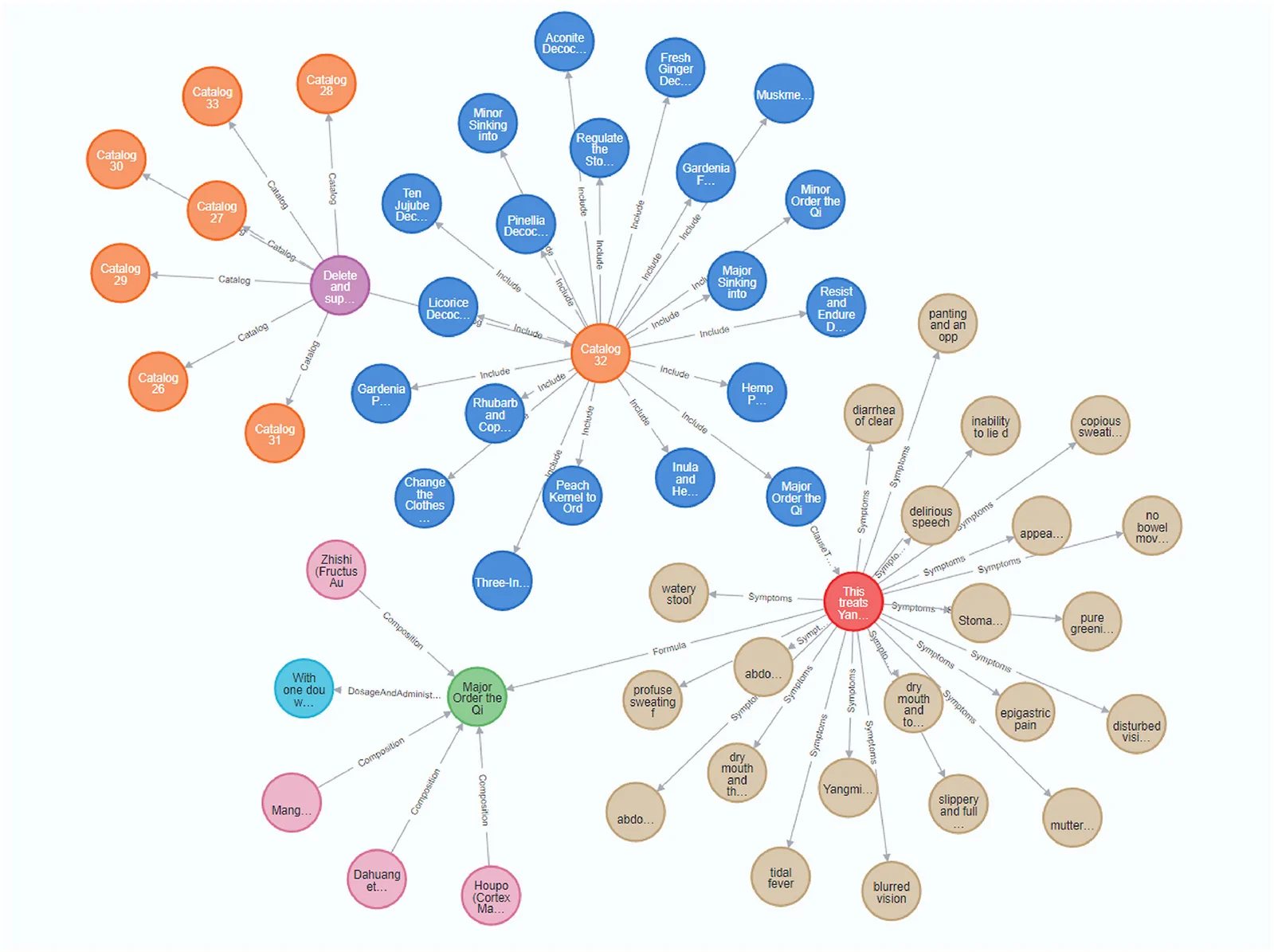
Partial visualization of the constructed *Golden Mirror of Medicine* knowledge graph in Neo4j, showing representative entity types and semantic relations. The graph was generated from relational triples extracted from the source text with LLM assistance, manually verified by TCM professionals, and imported into Neo4j. Node colors denote different entity types: book titles (purple), catalogs (orange), chapters (dark blue), clause texts (red), symptoms (light brown), formulas (green), herbs (pink), and administration methods (light blue).

### 3.2 Model

We selected GLM-4-Flash (https://bigmodel.cn/) as the core of our educational system due to its advantages in Chinese language processing and accessibility. Foremost, it provides strong Chinese semantic understanding, which is crucial for accurately parsing the specialized terminology and classical literature inherent to TCM. The model also integrates seamlessly with our specialized TCM knowledge graph via the LangChain library (https://www.langchain.com/). This synergy enables the system to leverage structured knowledge for more precise reasoning and retrieval, thereby supporting the generation of structured and context-relevant educational content. Finally, its computational efficiency and accessibility offer practical benefits for educational applications. These characteristics make GLM-4-Flash a suitable foundation for our Intelligent TCM Tutoring System.

### 3.3 Overall architecture of the Intelligent TCM Tutoring System

The Intelligent TCM Tutoring System, based on LLMs and KGs, employs a three-tier architecture—data, architecture, and application layers—to ensure scalability and interoperability (see Figure 3). The Data Layer constructs a structured knowledge base by integrating domain-specific texts (e.g., *Golden Mirror of Medicine*), LLMs, and human validation. Raw text is converted into relational triples via an LLM-driven, human-verified process and stored in a Neo4j graph database. This approach ensures semantic integrity and relational traceability, providing high-quality knowledge for upper-layer functions. The Architecture Layer manages user-resource interactions. It uses an intent recognition algorithm to parse user queries, leveraging the LLM for semantic analysis and Neo4j for precise KG retrieval and reasoning. A modular tool-calling mechanism enables flexible task execution, such as generating task-specific content by combining semantic understanding with graph path analysis. The Application Layer delivers educational services across pre-class, in-class, and after-class stages. Functionally, it comprises three core modules:

**Figure 3:**
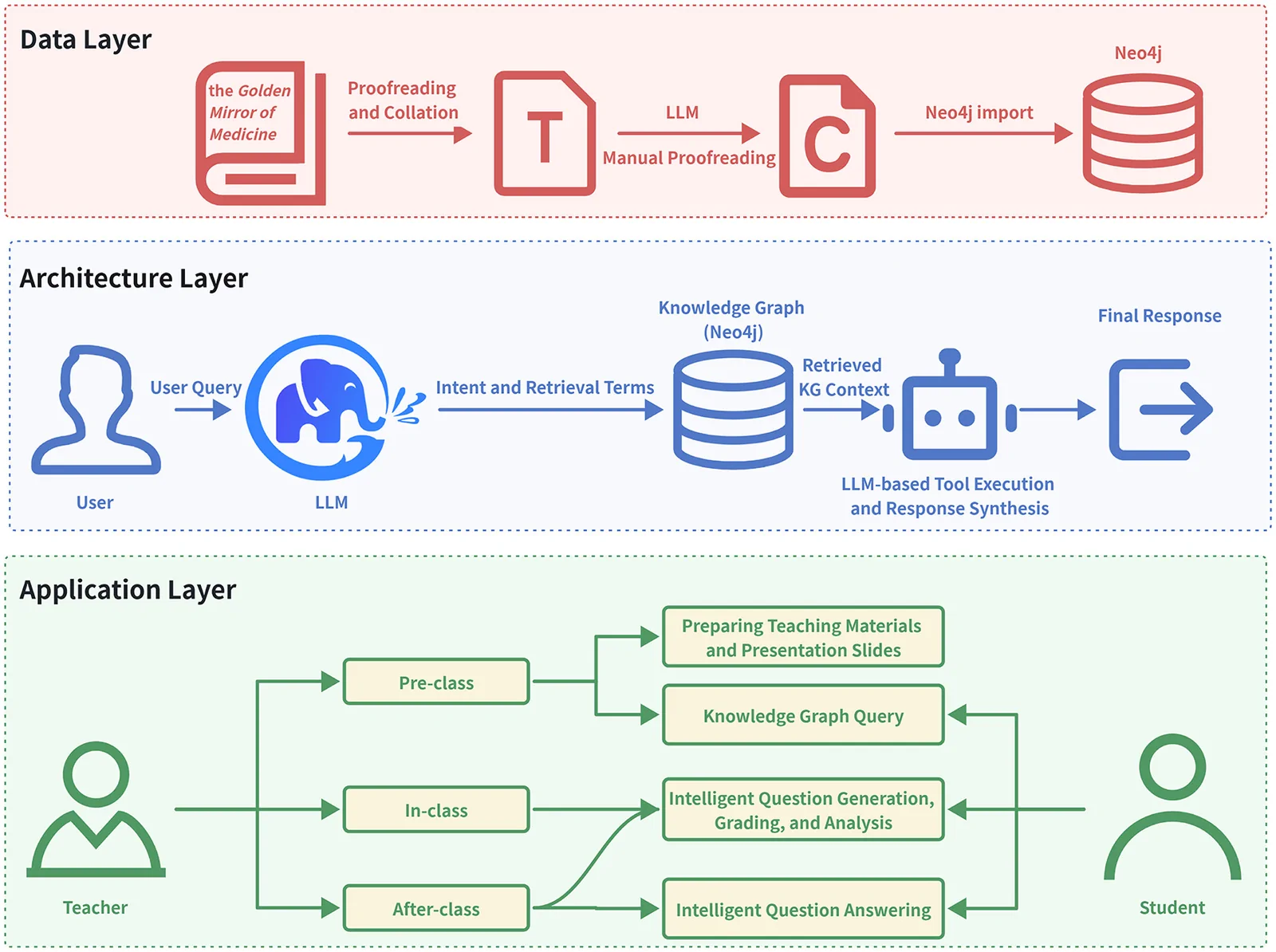
Three-layer architecture and end-to-end workflow of the Intelligent TCM Tutoring System integrating a large language model (LLM) with a knowledge graph (KG). The data layer summarizes source-text collation, LLM-assisted relation extraction, manual verification, and Neo4j storage. The architecture layer traces a user query through LLM-based intent processing, KG retrieval, response synthesis, and final output. The application layer presents the teacher- and student-facing functions across pre-class, in-class, and after-class activities.

Instructional content generation: automatically creates syllabi, schedules, and presentation slides, and facilitates KG-based exploration to aid in teaching preparation and structured learning.

Intelligent question answering (Q&A): provides query-specific answers generated by the LLM and grounded in the KG, promoting knowledge consolidation.

Automated test generation and grading: supports real-time formative assessment during class and summative assessment after class with automatic test creation, grading, and detailed feedback.

#### 3.3.1 The Instructional Content Generation Module

The Instructional Content Generation Module automates the creation of teaching syllabi, instructional schedules, and presentation slides to meet pedagogical needs. By leveraging an LLM, the module analyzes instructor-uploaded e-textbooks (PDF format) to generate a structured teaching plan detailing weekly topics, learning objectives, and instructional requirements. The process invokes the LLM's API to extract and process data; the extracted data then populate predefined templates. The system produces editable Word documents for syllabi and schedules, and caches files to ensure fault tolerance. Furthermore, it can generate presentation slides from specified knowledge points, reducing instructor preparation time. The module aims to enhance instructional efficiency and quality, facilitate flexible content delivery, and support student learning.

#### 3.3.2 The Intelligent Q&A Module

The Intelligent Q&A Module, a core system component, integrates a KG with an LLM to provide real-time responses to student inquiries. Upon receiving a query, the system extracts keywords, retrieves relevant knowledge from the KG, and then employs the LLM to synthesize a precise natural-language answer. This KG-grounded approach enhances Q&A efficiency and facilitates deeper learning by accurately addressing complex theoretical questions in TCM.

#### 3.3.3 The Automated Test Generation and Grading Module

The Automated Test Generation and Grading Module streamlines the assessment lifecycle, from item creation to feedback delivery. Leveraging the instructional content from e-textbooks, the system uses an LLM to generate test items (including questions and standard answers) relevant to specific courses and chapters. Instructors can then compile and distribute these tests via the user interface. Upon submission, the system performs automated grading, including LLM-powered scoring of questions by comparing student responses to standard answers. Crucially, the module also generates detailed explanations for incorrect answers, providing students with immediate, formative feedback to improve their comprehension and learning outcomes.

### 3.4 Interface of the Intelligent TCM Tutoring System

The system interface is primarily divided into two parts: the teacher terminal and the student terminal, each presenting functionalities tailored to its respective user group.

#### 3.4.1 The user interface of the student terminal

Figure 4 shows the student interface of the Intelligent TCM Tutoring System, designed with a student-centered philosophy. The interface is structured into three modules that mirror the complete learning workflow: “Pre-class Preview,” “Learning in class,” and “After-class consolidation,” supporting systematic learning across different stages.

**Figure 4:**
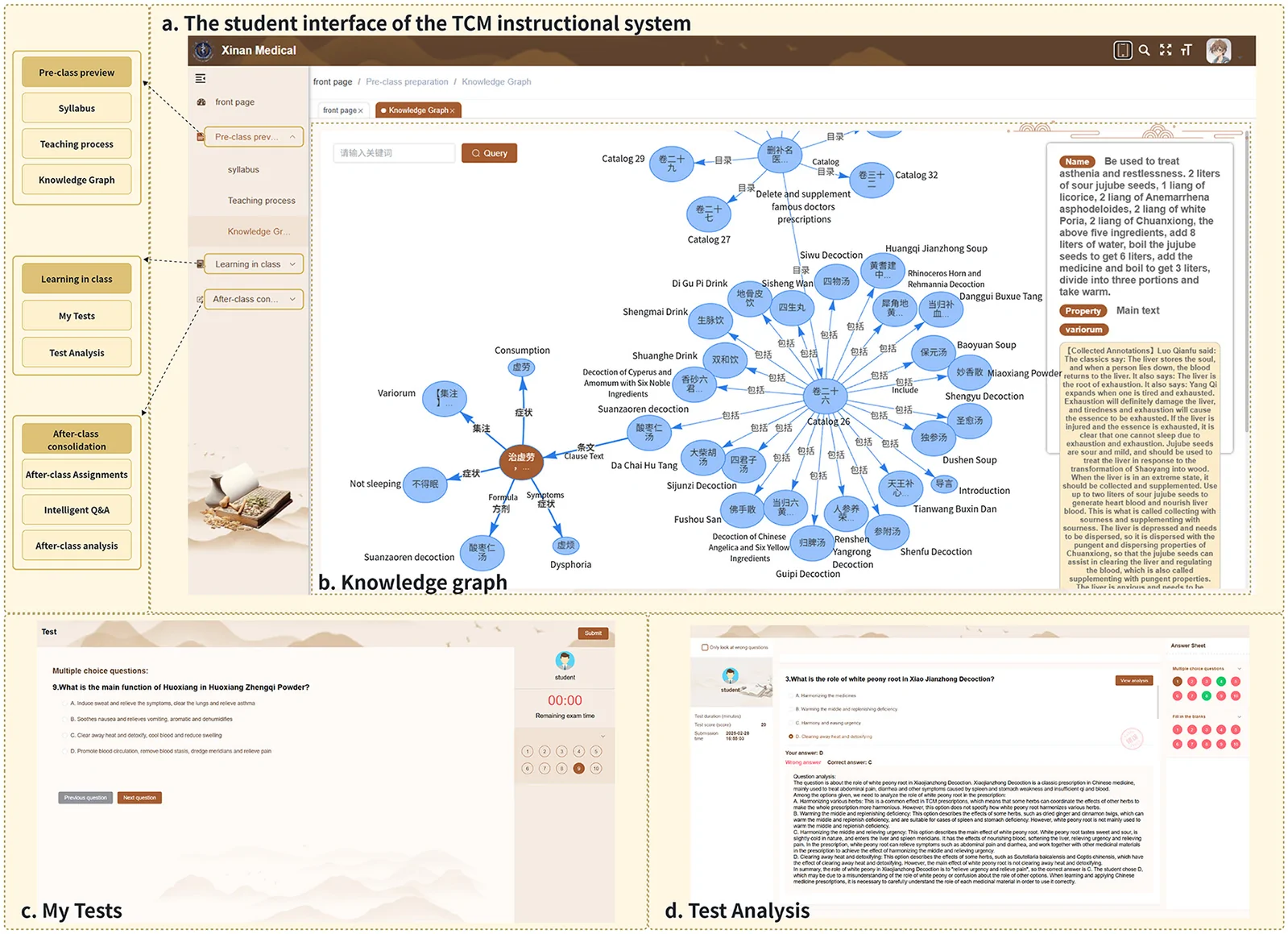
Screenshots from the implemented student interface, illustrating how the system supports learning across pre-class, in-class, and after-class stages. **(a)** The student-side navigation structure, including Pre-class Preview, Learning in Class, and After-class Consolidation. **(b)** The interactive knowledge graph, in which selecting a node displays its associated information. **(c)** The “My Tests” module for completing assigned assessments. **(d)** The “Test Analysis” report presenting item-level answers, results, and explanations.

The “Pre-class Preview” module includes a Syllabus, Teaching Schedule, and a KG. The KG visually represents the course's knowledge structure, allowing users to explore interconnected concepts by clicking on nodes to retrieve detailed information, such as content, annotations, and related TCM formulas. This feature is designed to foster a systematic understanding. The “Learning in class” module offers “My Tests” for immediate knowledge consolidation and “Test analysis” for detailed feedback. The analysis function provides correct answers and rationales for incorrect responses, helping students identify knowledge gaps. The “After-class consolidation” module, featuring After-class Assignments and Intelligent Q&A, supports extended practice and review. While functionally similar to the in-class module, its pedagogical focus shifts from formative assessment to in-depth mastery. Collectively, these integrated modules provide holistic support throughout the learning process, guiding students from initial preparation to deep consolidation.

#### 3.4.2 The user interface of the teacher terminal

The teacher terminal of our Intelligent TCM Tutoring System (Figure 5) is designed based on the principles of an “integrated teaching process” and “human-computer collaborative enhancement.” It organizes the entire teaching workflow into four core modules: Yizong Q&A, Preparation before class, In-class Teaching, and After-class Services.

**Figure 5:**
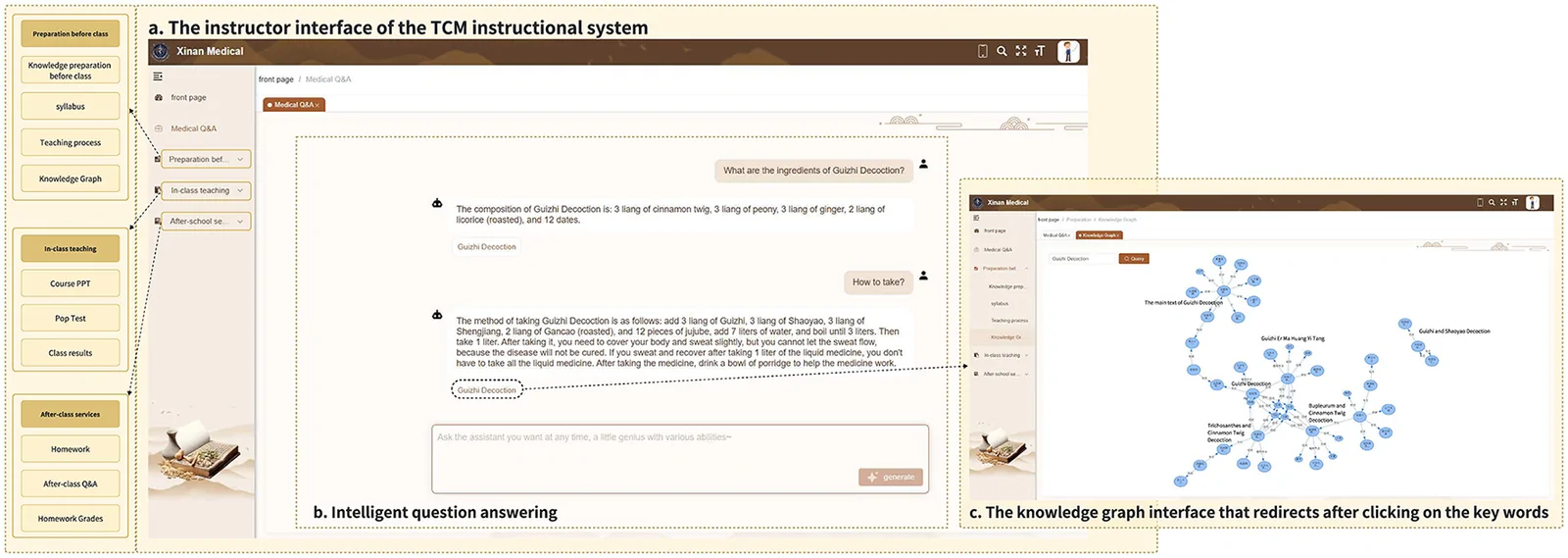
Screenshots from the implemented instructor interface, illustrating the teacher-facing instructional workflow and knowledge-graph-linked question answering. **(a)** The instructor-side navigation structure organized around Preparation before class, In-class Teaching, and After-class Services. **(b)** The intelligent question-answering module, in which generated answers include clickable links to relevant entities in the knowledge graph. **(c)** The knowledge-graph visualization opened by selecting a linked entity in the generated answer.

The Yizong Q&A module features an intelligent engine that uses NLP for semantic analysis of queries about the *Golden Mirror of Medicine*. It provides text-based answers linked to relevant KG nodes, enhancing response explainability and allowing for deeper exploration. The “Preparation before class” module facilitates resource organization and planning through functions such as structured management of uploaded textbooks, standardized generation of syllabi and schedules, and KG visualization to help instructors build a coherent knowledge framework. To enhance classroom interaction and formative assessment, the “In-class Teaching” module supports automated generation of presentation slides from keywords, customizable in-class tests, and real-time grading with feedback. This enables teachers to dynamically adjust their instruction. Subsequently, the “After-class Services” module supports summative assessment and instructional refinement. It includes tools for creating and grading homework, focusing on independent practice, and features an intelligent Q&A to assist teachers in preparing materials or refining their teaching strategies. This integrated design provides comprehensive support across the entire teaching lifecycle, augmenting the instructor's capabilities at each stage.

## 4 Results

This section outlines the experimental design for evaluating system performance and the impact on student learning by comparing the Intelligent TCM Tutoring System with GLM-4-Flash without KG integration.

### 4.1 Evaluation of system performance

System performance was assessed using the Accuracy, Relevance, and Smoothness (ARS) evaluation method. Five TCM teachers with prior experience in teaching the *Golden Mirror of Medicine* were selected as evaluators. None of the evaluators had participated in the development of the system.

The teachers assessed the instructional materials generated by the system across different modules, including the Instructional Content Generation Module, the Intelligent Q&A Module, and the Automated Test Generation and Grading Module. The five teachers reviewed the generated content, focusing on informational accuracy, relevance to the textbook, and linguistic smoothness. In the Instructional Content Generation Module, the generated presentation slides were evaluated; since the native GLM-4-Flash model cannot generate presentation slides, it was instructed to generate only the text content for comparison. For the Intelligent Q&A Module, a list of 20 questions covering the content of the *Golden Mirror of Medicine* was prepared for evaluating the generated answers. In the Automated Test Generation and Grading Module, the assessment focused on the generated exam papers and their corresponding question analyses.

All five evaluators received the same evaluation materials and a standardized ARS assessment form with identical scoring criteria and completed their assessments independently. The ARS evaluation used a 3-point scoring scale, with higher scores indicating better system performance. The scoring criteria are defined as follows. A score of 1 indicates that the generated content is completely unacceptable because of serious issues such as factual errors, incompleteness, or logical inconsistencies. A score of 2 signifies that the content is largely acceptable but requires some improvement. A score of 3 represents high-quality content that effectively supports the overall teaching objectives. The evaluation results are presented in Table 1. For the inter-rater reliability analysis, each evaluator's item-level scores were averaged separately by system module, evaluated system, and ARS dimension, following the structure presented in Table 1. The reliability of the mean ratings across the five evaluators was assessed using a two-way random-effects, absolute-agreement, average-measures intraclass correlation coefficient (ICC). The resulting ICC (A,5) was 0.847, indicating good inter-rater reliability.

**Table 1:** ARS assessment results.

Module	Model	Accuracy	Relevance	Smoothness
The Instructional Content Generation Module	GLM-4-Flash	2.18	2.24	2.34
	Intelligent TCM Tutoring System	2.82	**2.90**	**2.60**
The Intelligent Q&A Module	GLM-4-Flash	1.76	2.13	2.23
	Intelligent TCM Tutoring System	**2.77**	**2.82**	**2.82**
The Automated Test Generation and Grading Module	GLM-4-Flash	1.83	1.75	2.26
	Intelligent TCM Tutoring System	**2.92**	**2.88**	**2.84**

Bold values indicate the higher mean rating within each module and evaluation criterion.

The evaluation results indicated that the Intelligent TCM Tutoring System received higher ARS ratings than GLM-4-Flash across instructional content generation, intelligent Q&A, and automated test generation and grading.

In the Instructional Content Generation Module, the system received higher ratings than GLM-4-Flash for accuracy (2.82), relevance (2.90), and smoothness (2.60), demonstrating its ability to precisely generate teaching materials that are highly aligned with textbook content. Moreover, the Intelligent TCM Tutoring System generated structured textual content and automatically created supplementary teaching materials such as presentation slides, thereby supporting teaching efficiency. This content-generation capability may facilitate a more systematic and complete transfer of knowledge during the teaching process.

In the Intelligent Q&A Module, the Intelligent TCM Tutoring System received consistently high ratings. Its scores for accuracy (2.77), relevance (2.82), and smoothness (2.82) were higher than those of GLM-4-Flash, indicating its ability to generate accurate and relevant responses to TCM-related questions. The system provides precise answers that conform to medical theory and are logically structured and clearly expressed, thereby reducing factual and logical inconsistencies. This may help students obtain more reliable and coherent answers during self-study or when seeking assistance.

In the Automated Test Generation and Grading Module, the Intelligent TCM Tutoring System received high ratings for accuracy (2.92), relevance (2.88), and smoothness (2.84). The system generates test questions based on the teaching content and supports automated grading. It can automatically grade student answer sheets, potentially improving grading efficiency and supporting consistency in the process.

Overall, the expert ratings indicated that the Intelligent TCM Tutoring System produced accurate, relevant, and fluent outputs across the evaluated modules. In this TCM setting, the system generated instructional content, provided question-answering support, and created and graded test items. These findings support the system's potential as a supplementary tool for student learning and instructor-led teaching.

### 4.2 Evaluation of student learning effectiveness

To investigate the impact on student learning, we recruited 125 volunteers from Anhui University of Chinese Medicine to participate in an experiment. After recruitment and before the learning session, a researcher randomly allocated the 125 participants to the three study groups by drawing lots, resulting in initial group sizes of 43, 41, and 41, respectively. Group assignments remained concealed until each lot was drawn, preventing the researcher from influencing or selecting group assignments. All three groups studied the content of Volume 26 of the *Golden Mirror of Medicine*. Group 1 was provided with no Q&A support and relied entirely on self-study. Group 2 was provided with an LLM for Q&A, along with review questions and solutions generated by the LLM. Group 3 was provided with the Intelligent TCM Tutoring System, which integrates an LLM with a KG for Q&A, along with corresponding review questions and solutions. After completing the learning session, students' learning outcomes and experiences were assessed via a questionnaire featuring scales adapted from the work of Peng et al. (40).

The questionnaire consisted of four sections. The first section collected sociodemographic information. The second section was a scale measuring the overall learning experience, including engagement, perceived difficulty, and perceived performance. The third section, completed only by students in Groups 2 and 3, was a scale assessing the experience with the LLM and the Intelligent TCM Tutoring System, covering perceived usefulness, ease of use, and behavioral intention to use. The fourth section comprised seven test questions related to the learning content, with each question worth one point. The questionnaire included attention check questions to ensure data quality. The learning-experience scale items are detailed in Table 2. The questionnaire items were translated into Chinese by the research team and contextually adapted to refer to the learning content and systems used in the present study.

**Table 2:** Questionnaires used in the study.

Measurement	Items
Perceived engagement	Engagement (Cronbach's ***α*** **=** **0.796)** During this learning session, I was highly focused on learning the *Golden Mirror of Medicine*. During this learning session, I was so absorbed in learning the *Golden Mirror of Medicine* that I lost track of time. During this learning session, I invested a significant amount of effort in learning the *Golden Mirror of Medicine*.
Perceived difficulty	During this learning session, I found it very difficult to understand the content of the *Golden Mirror of Medicine*.
Perceived performance	**Understanding (Cronbach's** ***α*** **=** **0.951)** After this learning session, I am able to gain an in-depth understanding of the knowledge in the *Golden Mirror of Medicine* and analyze its practical applications. After this learning session, I am able to clearly articulate the key knowledge in the *Golden Mirror of Medicine* in my own words and explain its underlying logic. After this learning session, I am able to identify areas for further study within the *Golden Mirror of Medicine* and deepen my understanding of it using learning tools.
Frequency	I frequently used the Intelligent TCM Tutoring System to support my study of the *Golden Mirror of Medicine*.
Interruption	I found that the Intelligent TCM Tutoring System interrupted my learning process.
Technology acceptance	**Usefulness (Cronbach's** ***α*** **=** **0.945)** Using the Intelligent TCM Tutoring System enables me to understand the knowledge in the *Golden Mirror of Medicine* at a deeper level. Using the Intelligent TCM Tutoring System enhances my ability to master the knowledge in the *Golden Mirror of Medicine*. Using the Intelligent TCM Tutoring System made my learning of the *Golden Mirror of Medicine* more efficient. I find the Intelligent TCM Tutoring System highly useful for my study of the *Golden Mirror of Medicine*.
	**Ease of use (Cronbach's** ***α*** **=** **0.924)** I find the Intelligent TCM Tutoring System flexible to interact with. I find my interaction with the Intelligent TCM Tutoring System to be clear and straightforward. Using the Intelligent TCM Tutoring System to aid my study of the *Golden Mirror of Medicine* does not require significant mental effort. I find it easy to accomplish my learning tasks using the Intelligent TCM Tutoring System.
	**Intention to use (Cronbach's** ***α*** **=** **0.912)** If the Intelligent TCM Tutoring System were available to support my in-depth study of the *Golden Mirror of Medicine*, I would use it. In my future studies of the *Golden Mirror of Medicine* or other Traditional Chinese Medicine content, I plan to use the Intelligent TCM Tutoring System extensively.

All items were measured on a 7-point Likert scale (1 = Strongly Disagree; 7 = Strongly Agree). In the questionnaire, “intelligent teaching system” was used as an abbreviated reference to the Intelligent TCM Tutoring System. For students in Group 2, this term was correspondingly changed to “LLM”. Bold text indicates multi-item scales and their Cronbach's alpha coefficients.

Internal consistency was assessed using the final analyzed sample for each applicable scale. Cronbach's alpha values for all multi-item scales are reported in Table 2 (*n* = 102 for engagement and understanding; *n* = 68 for usefulness, ease of use, and intention to use, which were administered only to Groups 2 and 3). Cronbach's alpha was not applicable to the single-item measures. Construct validity was not assessed through factor analysis in the current study.

After collecting the questionnaire data from all groups, we conducted a statistical analysis of the differences among them. For comparisons involving the three groups, the Kruskal–Wallis *H*-test was used to assess overall differences, followed by pairwise comparisons with a Bonferroni correction to control for the risk of Type I errors associated with multiple comparisons. For comparisons involving only two groups, the Mann–Whitney *U*-test was employed for non-parametric statistical analysis. A two-sided *p*-value below 0.05 was considered statistically significant. This design aimed to systematically evaluate the effectiveness of different teaching methods and inform the further optimization of the Intelligent TCM Tutoring System. Given the non-normal distribution of the data, this study primarily reports medians and interquartile ranges (IQRs) to more accurately reflect the data's central tendency and dispersion. Additionally, to provide a comprehensive overview of the data, means and standard deviations (SDs) are also reported. Effect sizes were reported as epsilon-squared (ε^2^) for Kruskal–Wallis tests and rank-biserial correlation *r*_*rb*_ for Mann–Whitney *U*-tests. The 95% confidence intervals (CIs) for effect sizes were estimated using a non-parametric percentile bootstrap with 10,000 resamples performed separately within each group. Two-sided *p*-values were reported numerically, with values below 0.001 presented in scientific notation; pairwise *p*-values following Kruskal–Wallis tests were Bonferroni-adjusted. An approximate sensitivity analysis based on the final group sizes (34, 39, and 29), a two-sided α of 0.05, and 80% power indicated minimum detectable effect sizes of ε^2^ = 0.097 for the three-group comparisons and *r*_*rb*_ = 0.400 for the two-group comparisons involving Groups 2 and 3.

We tested the following hypotheses:

- H1: Learning outcomes. H1a: Post-test scores do not differ significantly among the three groups. H1b: Post-test scores differ significantly among the three groups, with the Intelligent TCM Tutoring System group expected to achieve higher scores than the other groups.
- H2: Learning experience. H2a: Learning-experience ratings do not differ significantly across the measured dimensions among the three groups. H2b: The Intelligent TCM Tutoring System group is expected to report more favorable ratings than both comparison groups for engagement, perceived difficulty, and perceived understanding. It is also expected to report more favorable ratings for frequency, interruption, perceived usefulness, usability, and acceptance than the LLM-assisted group.

During the data cleaning process, we excluded 23 questionnaires that failed attention checks or exhibited careless completion, yielding a final sample of 102.

#### 4.2.1 Descriptive statistical analysis

Table 3 presents the demographic characteristics and prior learning experience of the 102 participants included in the analysis across the three groups, including sex, age, major, and prior study of the *Golden Mirror of Medicine*.

**Table 3:** Participant characteristics across experimental groups.

**Characteristic**	Category	Group 1 (*n* = 34)	Group 2 (*n* = 39)	Group 3 (*n* = 29)	Total (*N* = 102)
Sex	Male	28 (82.4%)	30 (76.9%)	21 (72.4%)	79 (77.5%)
	Female	6 (17.6%)	9 (23.1%)	8 (27.6%)	23 (22.5%)
Age	18–25 years	24 (70.6%)	29 (74.4%)	22 (75.9%)	75 (73.5%)
	26–30 years	10 (29.4%)	10 (25.6%)	7 (24.1%)	27 (26.5%)
Major	TCM	8 (23.5%)	14 (35.9%)	8 (27.6%)	30 (29.4%)
	Non-TCM	26 (76.5%)	25 (64.1%)	21 (72.4%)	72 (70.6%)
Prior study of the *Golden Mirror of Medicine*	Yes	10 (29.4%)	10 (25.6%)	9 (31.0%)	29 (28.4%)
	No	24 (70.6%)	29 (74.4%)	20 (69.0%)	73 (71.6%)

*Values are presented as n (%), with percentages calculated within each group*.

Group-level comparisons of learning outcomes and learning experiences are summarized in Tables 4–6 and visualized in Figure 6.

**Figure 6:**
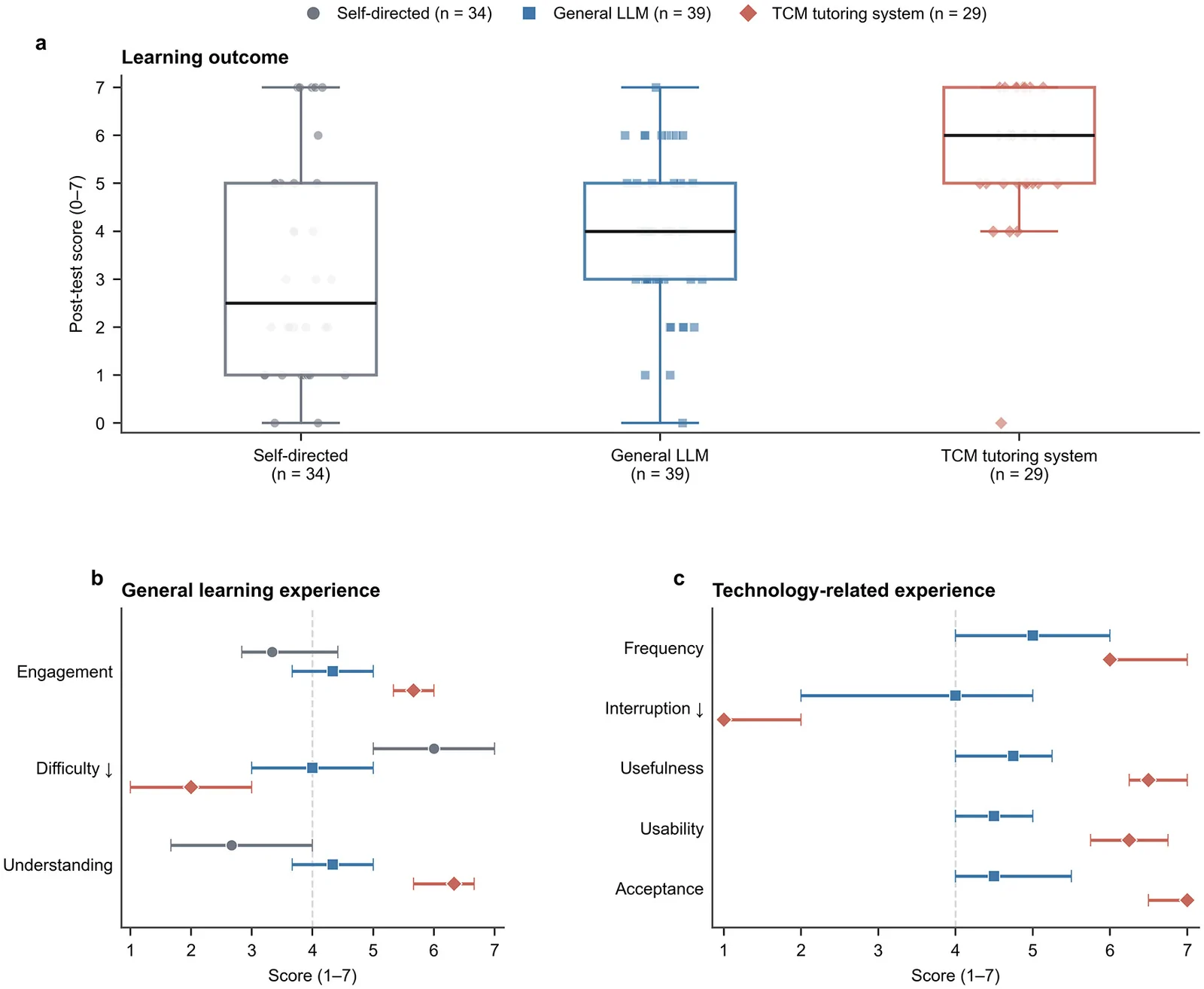
Group comparisons of learning outcomes and learning experiences. **(a)** Individual post-test scores for the self-directed learning (*n* = 34), LLM-assisted learning (*n* = 39), and Intelligent TCM Tutoring System-assisted (*n* = 29) groups; boxes show medians and interquartile ranges, whiskers extend to 1.5 times the interquartile range, and points represent individual participants. **(b)** Median (interquartile range) scores for engagement, difficulty, and understanding across the three groups. **(c)** Median (interquartile range) scores for frequency, interruption, usefulness, usability, and acceptance in Groups 2 and 3. Dashed lines in panels b and c indicate the midpoint of the 1–7 response scale. Lower scores indicate more favorable experiences for difficulty and interruption.

**Table 4:**
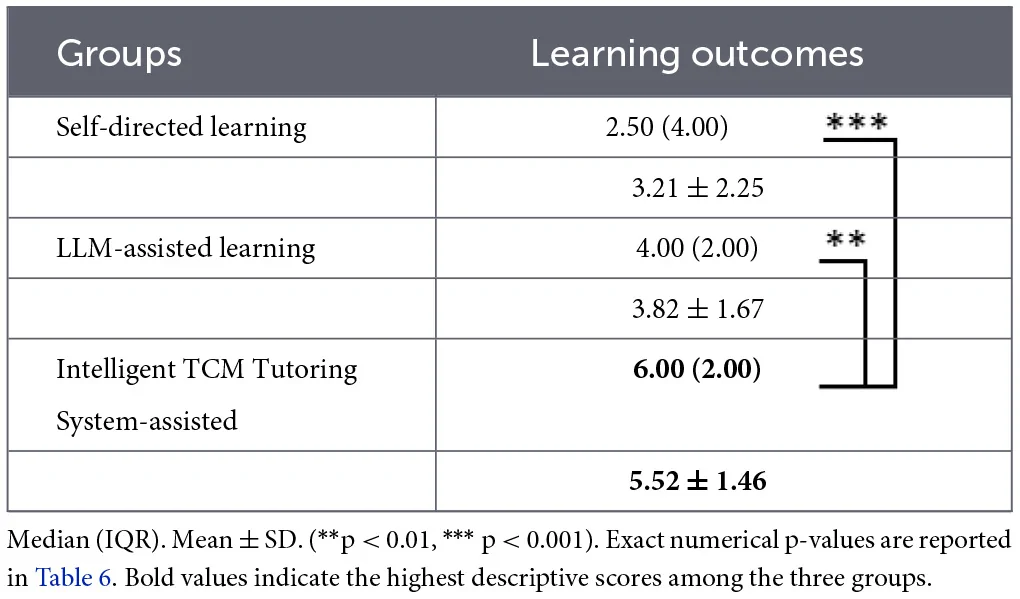
Learning outcomes by group.

Median (IQR). Mean ± SD. (^**^p < 0.01, ^***^ p < 0.001). Exact numerical p-values are reported in Table 6. Bold values indicate the highest descriptive scores among the three groups.

#### 4.2.2 Learning outcomes

Table 4 presents the learning outcome scores for three groups employing different learning methods. The self-directed learning group (Group 1) had a median score of 2.50 (IQR = 4.00), a mean of 3.21, and a standard deviation of 2.25. The LLM-assisted group (Group 2) had a median of 4.00 (IQR = 2.00), a mean of 3.82, and a standard deviation of 1.67. In contrast, the Intelligent TCM Tutoring System group (Group 3) had a median of 6.00 (IQR = 2.00), a mean of 5.52, and a standard deviation of 1.46. Statistical analysis revealed that the differences between Group 3 and both Group 1 and Group 2 were significant, with Group 3 demonstrating significantly higher learning outcomes than Group 1 and Group 2 (Bonferroni-adjusted *p* = 2.17 × 10^−5^ and *p* = 9.31 × 10^−4^, respectively). However, the difference between Group 1 and Group 2 was not statistically significant (Bonferroni-adjusted *p* = 0.861). These findings indicate that, in this single-session evaluation, the Intelligent TCM Tutoring System group achieved higher post-test scores than the self-directed learning and LLM-assisted groups.

These findings support H1b: post-test scores differed significantly among the three groups, and the Intelligent TCM Tutoring System group achieved higher scores than the other groups. Accordingly, H1a was not supported.

#### 4.2.3 Learning experience

Table 5 presents the evaluation results of the learning experience across different groups, covering multiple dimensions: Engagement, Difficulty, Understanding, Frequency, Interruption, Usefulness, Usability, and Acceptance. For the dimensions of Engagement, Difficulty, and Understanding, which involved comparisons among three groups, the Kruskal–Wallis test was employed to analyze overall differences, followed by *post-hoc* pairwise comparisons with Bonferroni correction; the reported *p*-values are Bonferroni-corrected. For the other dimensions, which involved comparisons between Groups 2 and 3, the Mann–Whitney *U*-test was used.

**Table 5:**
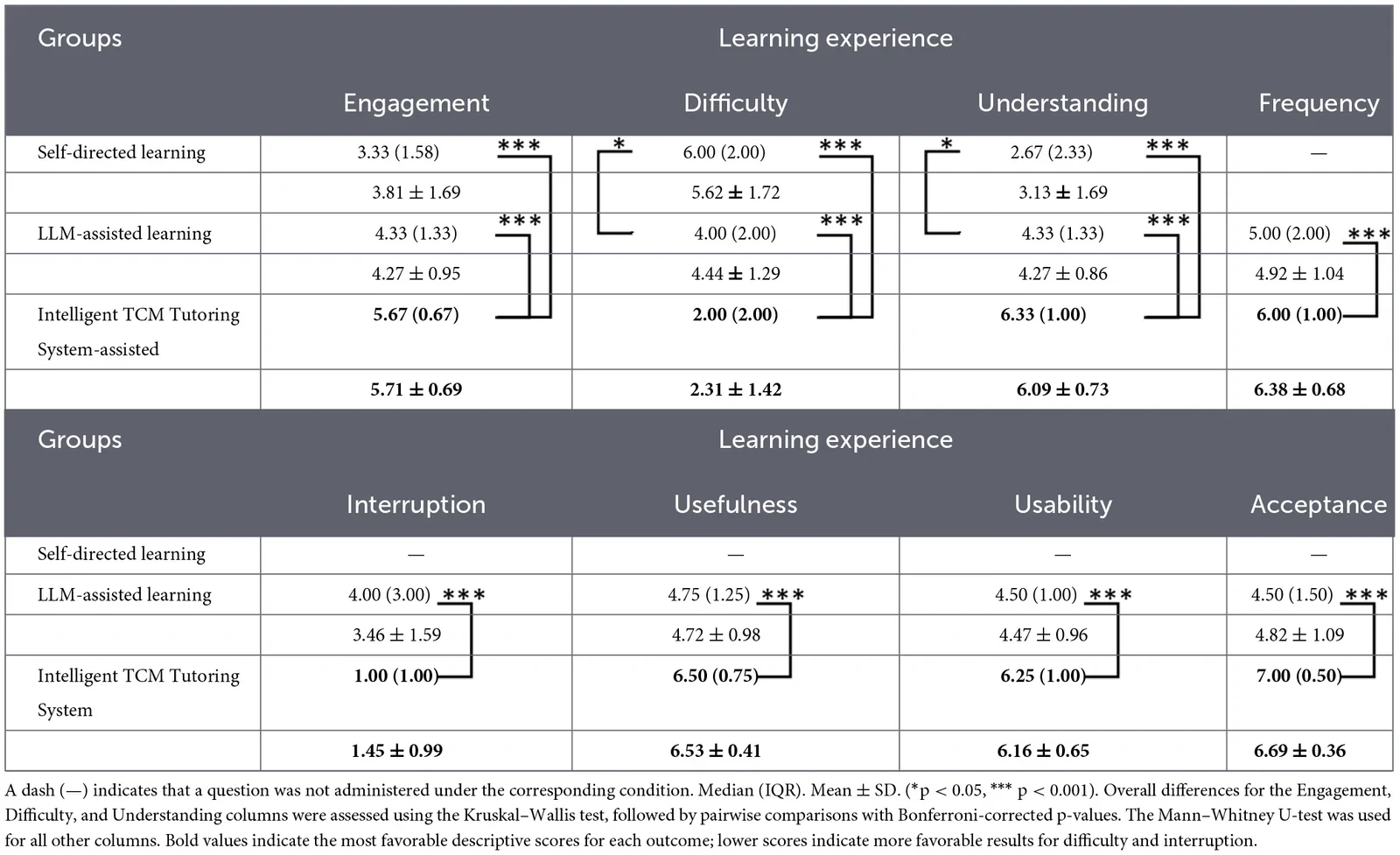
Learning experience by group.

A dash (—) indicates that a question was not administered under the corresponding condition. Median (IQR). Mean ± SD. (^*^p < 0.05, ^***^ p < 0.001). Overall differences for the Engagement, Difficulty, and Understanding columns were assessed using the Kruskal–Wallis test, followed by pairwise comparisons with Bonferroni-corrected p-values. The Mann–Whitney U-test was used for all other columns. Bold values indicate the most favorable descriptive scores for each outcome; lower scores indicate more favorable results for difficulty and interruption.

In terms of Engagement, the Intelligent TCM Tutoring System group (Group 3) scored 5.67 (IQR = 0.67), compared with 3.33 (IQR = 1.58) for the self-directed learning group (Group 1) and 4.33 (IQR = 1.33) for the LLM-assisted group (Group 2). The Kruskal–Wallis test indicated significant differences among the three groups (*p* = 3.41 × 10^−8^; ε^2^ = 0.327, 95% CI: 0.196–0.500). Bonferroni-adjusted pairwise comparisons showed that Group 3 had significantly higher engagement ratings than Group 1 (*p* = 7.15 × 10^−8^) and Group 2 (*p* = 1.27 × 10^−5^), whereas no significant difference was observed between Group 1 and Group 2 (*p* = 0.682).

Regarding Difficulty, the Intelligent TCM Tutoring System group had the lowest score (2.00, IQR = 2.00), compared with the LLM-assisted group (4.00, IQR = 2.00) and the self-directed learning group (6.00, IQR = 2.00). The Kruskal–Wallis test showed significant differences among the three groups (*p* = 1.58 × 10^−10^; ε^2^ = 0.436, 95% CI: 0.280–0.613). Bonferroni-adjusted pairwise comparisons indicated that Group 3 reported significantly lower difficulty ratings than Group 1 (*p* = 7.19 × 10^−1^^1^) and Group 2 (*p* = 4.77 × 10^−5^). The difference between Group 1 and Group 2 was also significant (*p* = 0.0217).

For Understanding, the Intelligent TCM Tutoring System group scored 6.33 (IQR = 1.00), compared with 2.67 (IQR = 2.33) for Group 1 and 4.33 (IQR = 1.33) for Group 2. Significant differences were observed among the three groups (Kruskal–Wallis test, *p* = 6.33 × 10^−12^; ε^2^ = 0.501, 95% CI: 0.353–0.666). Bonferroni-adjusted pairwise comparisons showed that Group 3 had significantly higher understanding ratings than Group 1 (*p* = 4.12 × 10^−12^) and Group 2 (*p* = 2.75 × 10^−6^). The difference between Group 1 and Group 2 was also significant (*p* = 0.0368).

For the dimensions administered only to Groups 2 and 3, the Mann–Whitney *U*-tests showed that the Intelligent TCM Tutoring System group reported more favorable ratings than the LLM-assisted group for frequency [6.00 (IQR = 1.00) vs. 5.00 (IQR = 2.00)], interruption [1.00 (IQR = 1.00) vs. 4.00 (IQR = 3.00)], usefulness [6.50 (IQR = 0.75) vs. 4.75 (IQR = 1.25)], usability [6.25 (IQR = 1.00) vs. 4.50 (IQR = 1.00)], and acceptance [7.00 (IQR = 0.50) vs. 4.50 (IQR = 1.50)]. Effect sizes and exact *p*-values for these comparisons are summarized in Table 6.

**Table 6:** Effect and *p*-values for group comparisons.

Outcome	Comparison/test	Effect size (95% CI)	*p*-value
Learning outcome	Three-group Kruskal–Wallis	ε^2^ = 0.201 (95% CI: 0.083, 0.377)	*p* = 1.74 × 10^−5^
Engagement		ε^2^ = 0.327 (95% CI: 0.196, 0.500)	*p* = 3.41 × 10^−8^
Difficulty		ε^2^ = 0.436 (95% CI: 0.280, 0.613)	*p* = 1.58 × 10^−10^
Understanding		ε^2^ = 0.501 (95% CI: 0.353, 0.666)	*p* = 6.33 × 10^−12^
Frequency	Group 3 vs Group 2, Mann–Whitney U	*r*_*rb*_ = 0.747 (95% CI: 0.576, 0.889)	*p* = 5.61 × 10^−8^
Interruption		*r*_*rb*_ = −0.708 (95% CI: −0.878, −0.508)	*p* = 2.67 × 10^−7^
Usefulness		*r*_*rb*_ = 0.877 (95% CI: 0.744, 0.976)	*p* = 6.65 × 10^−10^
Usability		*r*_*rb*_ = 0.872 (95% CI: 0.742, 0.972)	*p* = 8.91 × 10^−10^
Acceptance		*r*_*rb*_ = 0.825 (95% CI: 0.663, 0.954)	*p* = 4.08 × 10^−9^

CI, confidence interval; ε^2^, epsilon-squared; *r*_*rb*_, rank-biserial correlation. Positive *r*_*rb*_ values indicate higher scores in Group 3 than Group 2, whereas negative values indicate lower scores in Group 3. For three-group comparisons, p-values represent Kruskal–Wallis H-tests; Bonferroni-adjusted pairwise comparisons are described in the Results section.

In summary, the statistical analysis results support Hypothesis H2b: the Intelligent TCM Tutoring System group reported more favorable ratings than both comparison groups for engagement, difficulty, and understanding, and than the LLM-assisted group for frequency, interruption, usefulness, usability, and acceptance. In this single-session evaluation, the Intelligent TCM Tutoring System group reported more favorable post-session learning-experience ratings within the applicable group comparisons. Effect sizes for the learning-outcome and learning-experience comparisons are summarized in Table 6.

## 5 Discussion

Inspired by the principle of “teaching students in accordance with their aptitude,” this study examined how an integrated LLM–KG system can provide learner-responsive support in TCM education. The system combines language generation with structured TCM knowledge to provide KG-grounded explanations in response to students' queries and generate feedback on submitted answers. In the present implementation, adaptation is initiated through learner interaction rather than through an explicit learner model or automated longitudinal learning-path optimization.

Existing educational AI systems provide support through different mechanisms: Khanmigo emphasizes guided, inquiry-based interaction (28), GPT-based tools in medical education have been used for specific tasks such as standardized-patient simulation (26), and retrieval-augmented systems such as MedTutor (41) generate case-based materials from retrieved medical evidence. In contrast to these general-purpose or task-specific applications, the present system incorporates a human-validated KG derived from the *Golden Mirror of Medicine*, in which explicit relationships among diseases, symptoms, formulas, and medicinals support structured knowledge retrieval, exploration, and node-linked explanations. It further extends this domain grounding across an integrated instructional workflow encompassing content generation, intelligent question answering, assessment, grading, and feedback, rather than limiting KG support to a single educational task. Accordingly, this study contributes a domain-specific, human-validated LLM–KG framework and provides empirical evidence of its application in classical TCM education. The internal comparison with GLM-4-Flash without KG integration further provides context-specific evidence regarding the educational value of this integrated framework.

The system's performance was assessed using the ARS evaluation method. For the experiment, 125 student volunteers were recruited from Anhui University of Chinese Medicine. This experiment investigated the effects of three different learning modalities—self-directed learning, LLM-assisted learning, and learning with the Intelligent TCM Tutoring System based on the LLM and KG—on students' learning outcomes and experiences. A detailed analysis of each group's performance was then conducted.

To evaluate system performance, the ARS evaluation method was employed to compare the Intelligent TCM Tutoring System with GLM-4-Flash (without KG integration) across several modules: instructional content generation, intelligent Q&A, and automated test generation and grading. The results indicated that the Intelligent TCM Tutoring System received higher ARS ratings than GLM-4-Flash across all evaluated modules. In the Instructional Content Generation Module, the system received higher ratings for accuracy, relevance, and smoothness. The system generated instructional content aligned with the textbooks and also created supplementary teaching materials, such as presentation slides, which may support instructional efficiency. In the Intelligent Q&A Module, the Intelligent TCM Tutoring System received higher expert ratings for accuracy, relevance, and smoothness, indicating better performance in generating precise and coherent responses. Furthermore, in the Automated Test Generation and Grading Module, the Intelligent TCM Tutoring System also received higher expert ratings for accuracy, relevance, and smoothness across both test-item generation and automated grading.

Regarding student learning outcomes, the results for Hypothesis H1 showed that the Intelligent TCM Tutoring System group achieved significantly higher immediate post-test scores than the self-directed learning and LLM-assisted groups. These findings indicate better knowledge mastery immediately after the learning activity but do not establish sustained improvement in academic performance. Within this activity, the Intelligent TCM Tutoring System provided structured, domain-specific learning support. Although the LLM-assisted group scored higher than the self-directed learning group, the difference was not statistically significant, suggesting that standalone LLM support may be limited when structured and specialized knowledge support is absent.

In terms of learning experience, the results for Hypothesis H2 showed that the Intelligent TCM Tutoring System group reported significantly more favorable post-session ratings than both comparison groups for engagement, difficulty, and understanding. For frequency, interruption, usefulness, usability, and acceptance, the system group reported significantly more favorable ratings than the LLM-assisted group. In this post-session assessment, students in the system group reported higher engagement and perceived knowledge mastery, fewer interruptions, and higher usefulness, ease-of-use, and intention-to-use ratings. These findings indicate a more favorable immediate learning experience during the evaluated activity but do not establish sustained engagement. The LLM-assisted group performed well on some dimensions, such as engagement and comprehension, but its overall post-session ratings did not exceed those of the Intelligent TCM Tutoring System group. One possible explanation is the standalone LLM's lack of domain-specific knowledge support; integrating the LLM with a KG may have contributed to the more favorable outcomes observed after the activity.

Overall, the results of this single-session evaluation support Hypotheses H1b and H2b: the Intelligent TCM Tutoring System group achieved higher immediate post-test scores and reported more favorable post-session learning-experience ratings than the comparison groups. These findings provide preliminary evidence of the system's short-term educational value in this setting and should not be interpreted as evidence of sustained or long-term effectiveness. Furthermore, the system performance evaluation also showed higher expert ratings for the Intelligent TCM Tutoring System in its modules for instructional content generation, intelligent question-answering, and automated test generation and grading, supporting its potential application in TCM education. Together, these functions indicate that knowledge-grounded, learner-responsive support can supplement the instructor's guiding role.

## 6 Limitations and future directions

### 6.1 Limitations

Despite the positive findings of this study, several limitations should be acknowledged. First, the sensitivity analysis characterized the minimum detectable effect sizes under the final group sizes; however, the modest sample size may have limited the detection of smaller group differences. More broadly, this study provides preliminary empirical evidence on an integrated LLM–KG tutoring system in a defined TCM educational context; however, the external validity of these findings remains bounded by the study setting. The evaluation was conducted at a single Chinese university, used the *Golden Mirror of Medicine* as the sole domain source text, and examined a system based on one underlying LLM (GLM-4-Flash). Accordingly, the findings should not be assumed to generalize directly to other institutions, healthcare disciplines, cultural or linguistic settings, instructional sources, or AI models.

Second, learning outcomes and experiences were assessed only after a single learning session, and no delayed follow-up was conducted. Consequently, the study cannot determine long-term knowledge retention, sustained engagement, transfer to clinical or academic practice, or effectiveness over a longer educational period. Although major and prior study of the *Golden Mirror of Medicine* were available as limited indicators of TCM-related background, prior AI or LLM use, digital literacy, academic performance, and broader TCM knowledge were not measured; residual confounding by these characteristics therefore cannot be excluded. The construct validity of the adapted questionnaire was not independently established in this sample; therefore, the questionnaire dimensions should be interpreted cautiously and validated in larger independent samples. The current system did not maintain an explicit learner model or automatically adapt longitudinal learning pathways from individual performance data; accordingly, the present findings concern learner-responsive educational support rather than fully automated personalized learning.

### 6.2 Future directions

Future research should assess these participant characteristics prospectively and use multi-institutional, larger, and more diverse samples. It should also evaluate additional instructional sources and healthcare disciplines across cultural and linguistic settings and compare systems based on different LLMs. Delayed post-tests and longer-term follow-up in real-world instructional settings are also needed to examine knowledge retention, sustained engagement, transfer, longer-term effectiveness, and user acceptance.

Furthermore, as technology continues to advance, the functionality and design of the Intelligent TCM Tutoring System are amenable to continuous improvement. Future studies could integrate more advanced AI models with enhanced reasoning capabilities to more effectively emulate the decision-making processes of human teachers, thereby improving the flexibility and intelligence of pedagogical responses. An intelligent recommendation module could also be developed to dynamically identify and analyze students' knowledge gaps during the learning process. This module would then proactively deliver relevant learning resources and personalized guidance, thereby providing more precise learning path recommendations and personalized interventions to improve both the user experience and learning outcomes. Additionally, the potential application of the system in other academic disciplines could be explored to validate its cross-disciplinary applicability. This would not only enhance the technology's versatility but also provide innovative solutions for intelligent education across various fields.

## Data Availability

The datasets presented in this article are not readily available because data will be made available on reasonable request. Requests to access the datasets should be directed to Jili Hu, hujili@ahtcm.edu.cn.

## References

[1] Fu R Li J Yu H Zhang Y Xu Z Martin C. The Yin and Yang of traditional Chinese and western medicine. Med Res Rev. (2021) 41:3182–200. doi: 10.1002/med.21793 33599314

[2] Yuan C Zhang W Wang J Huang C Shu B Liang Q . Chinese medicine phenomics (chinmedphenomics): personalized, precise and promising. Phenomics. (2022) 2:383–8. doi: 10.1007/s43657-022-00074-x 36939806 PMC9712866

[3] Li N Wang T Xue X Wu X Zhao Y Zhao L. Thought on the integration of apprentice education and college education in the talents cultivation in TCM. World J Integr Tradit West Med. (2019) 14:1612–5. doi: 10.13935/j.cnki.sjzx.191132

[4] Wang F Zhang H. The important value and practical paths of master-apprentice education in the inheritance and innovation of traditional Chinese medicine culture. J Tradit Chin Med (2023) 64:1513–8. doi: 10.13288/j.11-2166/r.2023.15.001

[5] Tian J. Individualized teaching from ancient academies and personalization of modern higher education. Univ Educ Sci. (2020):94–101. doi: 10.3969/j.issn.1672-0717.2020.06.13

[6] Liu T. Guidance and respect: the meaning and path of teaching students in accordance with their aptitude from the analects of confucius. Guizhou Soc Sci. (2022):103–8. doi: 10.13713/j.cnki.cssci.2022.06.004

[7] Shah Ph D Kumar R. Concepts of learner-centred teaching. Shanlax Int J Educ. (2020) 8:45–60. doi: 10.34293/education.v8i3.2926

[8] Cheng L. “Changes” and “Constants”: rational reflection on large-scale individualized teaching in the age of digital intelligence. e-Education Res. (2025) 46:34–9 + 46. doi: 10.13811/j.cnki.eer.2025.01.005

[9] Zhang S. Development model, external challenges, and future path exploration of Confucius institutes. J Sichuan Norm Univ. (2025) 52:137–45 + 205–6. doi: 10.13734/j.cnki.1000-5315.2025.0608

[10] Shen R Wang Y. Research on logic, problems and action paths of “Emerging Medical Education” construction. Med Philos. (2020) 41:69–73. doi: 10.12014/j.issn.1002-0772.2020.12.16

[11] Cui S Ma J Gao X Liu Z Liang H Bao H . The construction and practice of the “Theory - Classic - Clinical” three-in-one teaching system of basic theory of traditional Chinese medicine. J Li-shizhen Tradit Chin Med. (2022) 33:1737–9. doi: 10.3969/j.issn.1008-0805.2022.07.62

[12] Min L Lin X Liang W. Research on the optimization of teaching interaction in diagnosis of traditional Chinese medicine. J Li-shizhen Tradit Chin Med. (2022) 33:2757–8. doi: 10.3969/j.issn.1008-0805.2022.11.56

[13] Ren H Wang W Xu L Li H Jiang T Yang T . Thinking on the research of smart traditional Chinese medicine under the background of intelligent era. J Nanjing Univ Tradit Chin Med. (2024) 40:1291–302. doi: 10.14148/j.issn.1672-0482.2024.1291

[14] Yang T Ren H Zhou Z Zhu X Hu K. Issues and challenges in the AI-empowered high-quality development of traditional Chinese medicine. J Nanjing Univ Tradit Chin Med. (2024) 40:1285–1290. doi: 10.14148/j.issn.1672-0482.2024.1285

[15] Brown TB Mann B Ryder N Subbiah M Kaplan J Dhariwal P . Language models are few-shot learners. Advances in Neural Information Processing Systems Curran Associates, Inc. (2020) 33:1877–1901.

[16] Denny P Gulwani S Heffernan NT Käser T Moore S Rafferty AN . Generative AI for education (GAIED): advances, opportunities, and challenges. arXiv:2402.0158 (2024)

[17] Xu C. Intelligent recommendation method for digital teaching resources of online courses based on knowledge graph. IJCEELL. (2025) 35:62–76. doi: 10.1504/IJCEELL.2025.143802

[18] Ji S Liu L Xi J Zhang X Li X KLR-KGC: knowledge-guided LLM reasoning for knowledge graph completion. Electronics. (2024) 13:5037. doi: 10.3390/electronics13245037

[19] Wu Q. The Golden Mirror of Medicine (Volume 1, 2, 3). Beijing: People's Health Publishing House. (2017).

[20] Wang Y Wang Y Dong C Du J Sun J. Discussion on the feasibility of incorporating *Yizong Jinjian* into undergraduate teaching of traditional Chinese medicine. Chin Med Modern Distance Educ China. (2021) 19:17–8. doi: 10.3969/j.issn.1672-2779.2021.09.007

[21] Zheng X Yuan Y Han J. Clinical teaching enlightenment from the Golden Mirror of Medicine. Chin Med Modern Distance Educ China. (2023) 21:4–6. doi: 10.3969/j.issn.1672-2779.2023.06.002

[22] Ruan X. The design and implementation of the Golden Mirror of Medicine cultural town based on unity 3D (Master's thesis). Hunan University of Chinese Medicine (2023).

[23] Mihaljević B Radovan A Beronić D. Integrating AI-assisted learning of programming in software development courses. Seville, Spain (2024), p. 10273–83. doi: 10.21125/iceri.2024.2623

[24] Spina AC Fereydouni P Tang JN Andalib S Picton BG Fox AR. Tailoring glaucoma education using large language models: addressing health disparities in patient comprehension. Medicine. (2025) 104:e41059. doi: 10.1097/MD.0000000000041059 39792725 PMC11729625

[25] Guo Q Zhen J Wu F He Y Qiao C. Can students make STEM progress with the large language models (LLMs)? An empirical study of LLMs integration within middle school science and engineering practice. J Educ Comput Res. (2025) 63:372–405. doi: 10.1177/07356331241312365

[26] Wang C Li S Lin N Zhang X Han Y Wang X . Application of large language models in medical training evaluation—using ChatGPT as a standardized patient: multimetric assessment. J Med Internet Res. (2025) 27:e59435. doi: 10.2196/59435 39742453 PMC11736217

[27] Ye B Xi Y Zhao Q. Optimizing mathematical problem-solving reasoning chains and personalized explanations using large language models: a study in applied mathematics education. JAPMI. (2024) 3:67–83. doi: 10.60087/Japmi.Vol.03.Issue.01.Id.005

[28] Khan Academy. Meet Khanmigo: Khan Academy's AI-powered teaching assistant & tutor. https://www.khanmigo.ai/ (Accessed May 8, 2025).

[29] Hou B Lin Y Li Y Fang C Li C Wang X. KG-PLPPM: a knowledge graph-based personal learning path planning method used in online learning. Electronics. (2025) 14:255. doi: 10.3390/electronics14020255

[30] Wu W Yang S Tian F Wei X Pan Y. Knowledge graph enhanced recommendation for semantic structure construction and feedback in online learning. Phys Commun. (2025) 69:102590. doi: 10.1016/j.phycom.2024.102590

[31] Zhong Y Zhang X Su Y. Recommendation method of teaching resources for professional music courses based on knowledge graph. Int J Hi Spe Ele Syst. (2025) 34:2540212. doi: 10.1142/S0129156425402128

[32] Ramazanova V Sambetbayeva M Serikbayeva S Sadirmekova Z Yerimbetova A. Development of a knowledge graph-based model for recommending MOOCs to supplement university educational programs in line with employer requirements. IEEE Access. (2024) 12:193313–31. doi: 10.1109/ACCESS.2024.3519263

[33] Lu H Li X. Chinese language curriculum resources pushing method based on knowledge graph and similarity algorithm. IJCEELL. (2025) 35:77–94. doi: 10.1504/IJCEELL.2025.143804

[34] Villegas-Ch W Govea J Gutierrez R. Optimizing language model-based educational assistants using knowledge graphs: integration with Moodle LMS. IEEE Access. (2024) 12:191994–2012. doi: 10.1109/ACCESS.2024.3518952

[35] Bui T Tran O Nguyen P Ho B Nguyen L Bui T . Cross-data knowledge graph construction for LLM-enabled educational question-answering system: a case study at HCMUT. In: Proceedings of the 1st ACM workshop on AI-powered Q&A systems for multimedia. Phuket: ACM (2024). p. 36–43. doi: 10.1145/3643479.3662055

[36] Zhang X Zhang L Yan S Hou M Zhao Y. Personalized learning recommendation in collaboration of knowledge graph and large language model. J Comput Appl. 45:773–784. doi: 10.11772/j.issn.1001-9081.2024070971

[37] Liu J Sun X Zhang Y. Educational resource content review method based on knowledge graph and large language model collaboration. J East China Normal Univ. (2024):57–69. doi: 10.3969/j.issn.1000-5641.2024.05.006

[38] Liu X Tang C Xi C. Construction of an intelligent question-answering system for selected readings of the inner canon based on a multimodal knowledge graph. Educ Chin Med. (2025) 44:143–148. doi: 10.3969/j.issn.1003-305X.2025.01.438

[39] Institute Institute of Information on Traditional Chinese Medicine China China National Institute of Standardization. Health Informatics—Semantic Network Framework of Traditional Chinese Medicine Language System (2020). GB/T 38324-2019.

[40] Peng Z Liu Y Zhou H Xu Z Ma X. CReBot: Exploring interactive question prompts for critical paper reading. Int J Hum Comput Stud. (2022) 167:102898. doi: 10.1016/j.ijhcs.2022.102898

[41] Jang D Shangguan Z Tegtmeyer K Gupta A Czerminski JT Chheang S . MedTutor: a retrieval-augmented LLM system for case-based medical education. In:Habernal I Schulam P Tiedemann J, editors. Proceedings of the 2025 conference on empirical methods in natural language processing: system demonstrations. Suzhou: Association for Computational Linguistics (2025). p. 319–53. doi: 10.18653/v1/2025.emnlp-demos.24

